# Tumorigenic role of tacrolimus through mTORC1/C2 activation in post-transplant renal cell carcinomas

**DOI:** 10.1038/s41416-024-02597-8

**Published:** 2024-02-10

**Authors:** Dorottya Moldvai, Dániel Sztankovics, Titanilla Dankó, Enikő Vetlényi, Gábor Petővári, Ágnes Márk, Attila Patonai, Gyula Végső, László Piros, Ádám Hosszú, Judit Pápay, Ildikó Krencz, Anna Sebestyén

**Affiliations:** 1 Department of Pathology and Experimental Cancer Research, Semmelweis University, Üllői út 26., H-1085 Budapest, Hungary; 2 Department of Surgery, Transplantation and Gastroenterology, Semmelweis University, Üllői út 78., H-1082 Budapest, Hungary; 3 Department of Paediatrics (Bókay street Unit), Semmelweis University, Üllői út. 26, H-1085 Budapest, Hungary; 4 MTA-SE Lendulet Diabetes Research Group, Bókay János utca 53-54., H-1083 Budapest, Hungary

**Keywords:** Oncogenesis, Urological cancer

## Abstract

**Background:**

Kidney transplant recipients (KTRs) face an increased risk of renal cell carcinoma (RCC), in which the immunosuppressive regimen plays an important role. This study aimed to identify intracellular signalling alterations associated with post-transplant (post-tx) tumour formation.

**Methods:**

Expression of mTOR-related proteins were analysed in kidneys obtained from end-stage renal disease (ESRD) patients and RCCs developed in KTRs or non-transplant patients. The effects of tacrolimus (TAC) and rapamycin (RAPA) on mTOR activity, proliferation, and tumour growth were investigated through different in vitro and in vivo experiments.

**Results:**

Elevated mTORC1/C2 activity was observed in post-tx RCCs and in kidneys of TAC-treated ESRD patients. In vitro experiments demonstrated that TAC increases mTOR activity in a normal tubular epithelial cell line and in the investigated RCC cell lines, moreover, promotes the proliferation of some RCC cell line. In vivo, TAC elevated mTORC1/C2 activity in ischaemic kidneys of mice and enhanced tumour growth in xenograft model.

**Conclusions:**

We observed significantly increased mTOR activity in ischaemic kidneys and post-tx RCCs, which highlights involvement of mTOR pathway both in the healing or fibrotic processes of kidney and in tumorigenesis. TAC-treatment further augmented the already elevated mTOR activity of injured kidney, potentially contributing to tumorigenesis during immunosuppression.

## Background

Kidney transplantation improves survival and quality of life of patients with end-stage renal disease (ESRD). However, studies demonstrated an increased risk of cancer in kidney transplant recipients (KTR) compared to normal population [1]. The incidence of post-transplant (post-tx) tumours ranges around 7–33%, whereof renal cell carcinoma (RCC) is one of the most common types [2]. However, the characteristics and distribution of tumours vary both temporally and geographically [3]. Despite significant improvements in transplant care and oncology over the past decades, there has not been significant decrease in the incidence of post-tx cancer in KTRs [4].

There are several known risk factors involved in the development of post-tx RCC, such as the immunosuppressive (IS) regimen, recipient’s sex (male), higher age at transplant, race (lower hazard ratio for individuals of Asian compared to Caucasian and African descent) and prolonged duration of dialysis before transplantation. The indication for transplantation also affects the risk of developing post-tx cancer: glomerular disease, hypertensive nephrosclerosis and vascular diseases increase, while diabetes mellitus and tubular- or interstitial diseases decrease the risk [1]. In addition to the higher occurrence of cancer, cancer-specific mortality is also higher in KTRs than in non-transplant (non-tx) cancer patients. This can be due to the immunocompromised status caused by the maintenance IS therapy.

The most currently used maintenance IS agents have already been associated with an increased risk of developing tumours. The most commonly applied first-line IS drugs are the calcineurin-inhibitors (CNIs): cyclosporine A (CsA, FDA approved in 1983, used mainly until 1996) and its most widely used successor, tacrolimus (TAC, FDA approved in 1994, still in use). The tumorigenic effect of CsA has been confirmed in numerous studies [5, 6]. TAC has shown to be superior to CsA in improving graft survival, however, it also exhibited neoplastic effects. There is evidence regarding the topical use of TAC inducing squamous cell carcinoma of the penis and tongue [7] and can also be associated with an increased risk of lymphoma [8]. According to a recent meta-analysis, TAC is associated with similar or higher carcinogenicity compared to CsA [9, 10], while other studies have not found any correlation between TAC and cancer development [11]. However, it is important to note that these studies evaluated the topical use of TAC, where minimal systemic absorption occurs [12]. TAC may be carcinogenic in a dose-dependent manner, but the carcinogenic blood level can only be achieved with systemic therapy, not topical use.

The PI3K/Akt/mTOR pathway plays an important role in renal physiology, and its inhibitors have been used as immunosuppressants and antitumor agents for decades. Rapamycin (RAPA) was initially identified as an antifungal antibiotic in 1975, but its immunoinhibitory and antitumor activities have also been recognised for 40–50 years [13, 14]. RAPA was first approved in 1999 by the FDA and in 2000 by the EMA. mTOR regulates several intracellular processes, including cell growth and death, metabolism, and motility. Activation of mTOR signalling plays a crucial role in tumorigenesis of numerous malignancies. mTOR inhibitors have currently been used as therapeutic agents in advanced RCC [15], and as IS agents for more than two decades [16]. Increasing evidence suggests a link between the use of mTOR inhibitors (rapamycin, everolimus etc.) and a lower risk of overall malignancy among recipients [17, 18]. Additionally, mTOR inhibitors could improve cancer-free survival after transplantation and have a nephroprotective potential [19]. Analysing the risk of tumour types separately, RAPA significantly reduces the incidence of non-melanoma skin cancer and RCC, however, it increases the incidence of prostate cancer. Therefore, the role of RAPA in the risk of post-tx cancer development may depends on the tumour type. For that reason, switching to RAPA is recommended and considered safe for recipients with a history of cancer [20]. The most common issue with mTOR inhibitors is the occurrence of adverse effects leading to discontinuation (e.g. hyperlipidaemia, hypercholesterolaemia, hyperglycaemia, diarrhoea, anaemia etc.) [21].

Other commonly used IS drugs have also been associated with carcinogenic effects. Corticosteroids and azathioprine both tend to increase carcinogenesis in patients [22–24], but there are contradictions in the experimental data as well [25, 26]. Mycophenolate mofetil (MMF) and mycophenolic acid seem to demonstrate a lower risk of malignancies [27]. However, it has already been suggested that MMF may be associated with a higher incidence of Kaposi’s sarcoma [28].

Post-tx RCCs occur more frequently (90%) in the native kidney of KTRs, while in the renal allograft, it is far less common (around 10%) [29]. Previous studies have drawn attention to the altered distribution of RCC subtypes after transplantation compared to non-tx cases. The most common subtype of RCC is clear cell RCC (ccRCC), accounting for about 75% of all RCC cases, while the occurrence of non-tx papillary renal cell carcinoma (pRCC) cases is only around 10%. After transplantation, pRCC is usually overrepresented (around 50%) [30, 31].

Our study aimed to examine the intracellular alterations of tumour formation and the possible reasons for changes in subtype distribution of post-tx RCCs. We analysed the expression of mTORC1 and mTORC2-related proteins in RCCs developed in KTRs and non-tx patients. Additionally, we investigated the effects of TAC and mTOR inhibitors in in vitro experiments and different in vivo models (human xenograft mouse model, ischaemia-reperfusion model). Our results suggested that besides mTORC1, mTORC2 could also have a significant role in post-tx tumour development.

## Methods

### Patients’ characteristics

Post-tx RCCs (*n* = 44; developed in the remained kidney of recipients) and non-tx RCCs (*n* = 46; developed in patients with no IS treatment) were included in this study (Table 1). Tumours were rereviewed and reclassified according to the guideline of *WHO Classification of Tumours Editorial Board; Urinary and male genital tumours; Lyon (France): International Agency for Research on Cancer; 2022; (WHO classification of tumours series, 5th ed.; vol. 8)*. Tissues from patients with ESRD (*n* = 10) and normal kidneys (donor kidneys that were not considered acceptable for transplantation due to surgical reasons, *n* = 3) were also studied. The tissues were obtained by surgical resection at Semmelweis University (Budapest, Hungary) between 2000 and 2015. The archived tissue samples were used with the approval of the Hungarian Scientific Council National Ethics Committee for Scientific Research (No. 7/2006).

**Table 1:** Clinicopathologic characteristics of RCC patients.

	No. of cases (%)	
	Post-transplant RCCs (*n* = 44)	Non-transplant RCCs (*n* = 46)
Age (years, mean ± SD)	52 ± 12.79	60 ± 13.42
Gender		
Male	35 (80)	28 (61)
Female	9 (20)	18 (39)
Histology		
ccRCC	12 (27)	28 (61)
pRCC	28 (64)	18 (39)
ccpRCC^a^	4 (9)	0 (0)
Grade		
1	16 (36)	7 (15)
2	24 (55)	27 (59)
3	4 (9)	11 (24)
4	0 (0)	1 (2)
Immunosuppression		
Tacrolimus	22 (50)	–
Cyclosporin A	19 (43)	–
Other^b^	3 (7)	–

Cases that occurred at the Transplantation Clinic of Semmelweis University between 2000 and 2015 were collected.

^a^ccpRCC = clear cell papillary renal cell carcinoma; this group is not involved in further analyses of the study.

^b^One patient received azathioprine treatment and two patients were treated with mTORC1 inhibitor (sirolimus or combined tacrolimus + everolimus) because of previous malignancies.

### Tissue microarray construction

Tissue microarray blocks (TMAs) were constructed by punching out 2-mm cores (at least two cores per patient) from representative areas of the paraffin blocks containing the RCCs, ESRDs and normal kidney tissues and embedding them in paraffin blocks using TMA Master (3DHistech, Hungary). Representative areas were selected by a pathologist using haematoxylin-eosin sections.

### Immunohistochemistry

Immunohistochemistry (IHC) was performed on 4-µm-thick sections. After deparaffinization and endogenous peroxidase blocking, antigen retrieval was performed for 30 min (10 mM citrate pH 6.0) using a pressure cooker. Slides were incubated with primary antibodies: anti-p-(Ser2448)-mTOR (Cell Signaling; USA (MA); cat#2976; 1:100), anti-p-(Ser235/236)-S6 (Cell Signaling; cat#2211; 1:100), anti-Rictor (Bethyl; USA (TX); cat#A500-002A; 1:1000), and anti-PTEN (Cell Signaling; cat#9188; 1:100) followed by Novolink Polymer (Leica Biosystems, Germany) or Vectastain Universal Quick HRP Kit (Vector Laboratories; USA) detection systems. 3,3’-Diaminobenzidine (DAB, Dako; Denmark) was used as chromogen with haematoxylin counterstaining. Immunostained sections were scanned and reviewed by two investigators using SlideViewer 2.7 software (3DHistech, Hungary). Immunoreactivity was assessed in carcinoma cells and tubular epithelial cells of normal kidneys and ESRDs. H-score was calculated by a semi-quantitative assessment of both the intensity of staining (0, 1+, 2+ or 3+) and the percentage of immunopositive cells, as previously described [32]. For each stain, median H-score values were identified (pmTOR: 130; pS6: 110; Rictor: 100) and categorised cases as “low” or “high” based on these values. Subsequently, contingency tables and chi-square tests were used to compare the distribution of “low” and “high” cases for each staining in the post-tx and non-tx cases, with a significance level set at *p* < 0.05. The activity of mTORC1 and mTORC2 was estimated based on the expression of p-mTOR (an active form of the catalytic subunit of mTORC1 and mTORC2), p-S6 (a downstream target of mTORC1) and Rictor (scaffold protein of mTORC2). Cases with high levels of both pmTOR and Rictor were considered to have high mTORC2 activity.

### Cell cultures and reagents

RCC primarily arises from proximal tubular epithelial cells;[33] therefore, a human immortalised proximal tubular epithelial cell line (HK-2) was used as control. HK-2 (ATCC; USA (VA); #CRL-2190) and RCC cell lines 786-O (ATCC; #CRL-1932) and ACHN (Sigma-Aldrich; USA (MA); #88100508) were cultured and treated in DMEM high glucose medium (Biosera, France), while A498 (ATCC; #HTB-44) cells were maintained in MEM medium (Sigma-Aldrich) at 37 °C, 5% CO_2_. Media were supplemented with 10% foetal bovine serum (FBS, Biosera), 2 mM L-glutamine (Biosera), and 100 UI/ml penicillin-streptomycin (Biosera). Cells were plated in 96-well plates (Sarstedt; Germany; 3000 cell/well) or into T25 flasks (Sarstedt; 2.5 ×10^4^ cells per flask) and after 24 h, the following treatments were added in refreshed media for 72 h: tacrolimus (TAC; 10 and 50 ng/mL; Merck; USA (NJ)), rapamycin (RAPA; 10 and 50 ng/mL; Merck) and PP242 (1 μM; Tocris; UK). The applied concentrations were selected based on the recommended IS serum levels [34, 35] and our previous studies [36–38]. Long-term TAC treatment (10 ng/mL; 21-day) was also applied in all cell lines. Cells were harvested every 3 to 4 day, and TAC was supplemented immediately after seeding.

### Western blot analysis

Cells were washed with phosphate-buffered saline (PBS) and lysed with lysis buffer (50 mM Tris, 10% glycerol, 150 mM NaCl, 1% Nonidet-P40, 10 mM NaF, 1 mM PMSF, 0.5 mM NaVO_3_, pH 7.5). Protein concentrations were quantified using the Bradford method (BioRad; USA (CA)), and proteins were separated by SDS-PAGE and blotted onto PVDF membranes. Membranes were incubated with primary antibodies: anti-p-(Ser235/236)-S6 (1:1000; #4858; Cell Signaling), anti-S6 (Cell Signaling; #2317; 1:1000), anti-p-(Ser473)-Akt (Cell Signaling; #4060; 1:1000), anti-pan-Akt (Cell Signaling; #4691; 1:1000), anti-p-(Ser2448)-mTOR (Cell Signaling; #2971; 1:1000), anti-mTOR (Cell Signaling; #2983; 1:1000), anti-Rictor (Cell Signaling; #2140; 1:1000), anti-Raptor (Abcam; UK; #ab40768; 1:1000) and anti-β-actin (Sigma-Aldrich; #A2228; 1:5000) as loading control, followed by biotinylated secondary antibodies and avidin-HRP complex (Vectastain Elite ABC HRP Kit; Vector Laboratories; USA (CA)). Membranes were developed with ECL reagent (Pierce ECL Western Blotting Substrate; Thermo Fisher Scientific; USA (MA)) using C-DiGit Blot Scanner (LI-COR Biosciences; USA (NE)). Protein expressions were quantitated by densitometry using Image Studio software (LI-COR Biosciences) and normalised by comparison with β-actin. After normalisation, phosphorylated/total protein ratio was calculated for phosphoproteins to characterise mTOR kinase (p-mTOR/mTOR), mTORC1 (p-S6/S6), and mTORC2 (p-Akt/pan-Akt) activity. The ratio of the scaffold proteins Raptor (mTORC1) and Rictor (mTORC2) was used to assess the relative amount of mTORC1 and mTORC2. All proteins were run on a Western blot at least twice or three times.

### Alamar Blue and sulforhodamine B assays

After the 72 h treatment, cell viability and proliferation were measured with Alamar Blue (AB) and sulforhodamine B (SRB) assays. AB (Thermo Fisher Scientific) was diluted 1:10 in the culture media, and fluorescence was measured after 4-hour using Fluoroskan Ascent FL fluorimeter (570–590 nm; Labsystems International; India) and evaluated by Ascent software (Labsystems International). For SRB assay, treated cells were fixed by cold 10% trichloroacetic acid (60 min; 4 °C), then washed with water and dried. After drying, cells were incubated with SRB (Sigma-Aldrich; USA; 0.4%; 15 min; RT; dissolved in 1% acetic acid). After washing with 1% acetic acid, the protein-bound dye was dissolved in 10 mM Tris. The absorbance was measured at 570 nm using a LabSystems Multiskan RC/MS/EX Microplate Reader (Labsystem International). Each measurement was performed sixfold and repeated three times. Relative cell proliferation was calculated as the percentage of untreated cells.

### Renal ischaemia reperfusion mouse model

8-week-old male C57BL/6 mice (*n* = 24) were used for renal ischaemia reperfusion (IR) mouse model. Animals were kept in cages with 5 animals per cage (12/12 light/dark; 24 °C; ad libitum food and water). Animals were randomised (simple method) to the following groups: #1: TAC (Advagraf; Astellas Pharma; in saline; 3 mg/kg/day; IP; IR surgery; *n* = 4); #2: IR IP control (saline; IP; IR surgery; *n* = 4); #3: SHAM IP control (saline; IP; placebo surgery; *n* = 4) #4: RAPA (Rapamune; Pfizer; 1.5 mg/kg/day; *per os*; IR surgery; *n* = 4); #5: IR *per os* control (saline; *per os*; IR surgery; *n* = 4); #6: SHAM *per os* control (saline; *per os*; placebo surgery; *n* = 4). In accordance with the 3 R (replacement, reduction, refinement) strategy, the group size was reduced as much as possible. The surgery was performed as the following: body temperature was maintained at 37 °C on a heating pad throughout anaesthesia (ketamine-xylazine; Sigma-Aldrich; USA). Renal ischaemia was accomplished by cross clamping the left renal artery and vein for 20 min with an atraumatic vascular clamp. Before the end of ischaemia, the contralateral kidney was removed, the clamp was withdrawn, and reperfusion was visually confirmed. SHAM animals underwent placebo surgery (got anaesthesia and opening the abdominal cavity without cross-clamping the artery and removing the kidney). Treatment protocol began 24 h after surgery. The required dose of TAC (3 mg/kg/day) was determined by blood level remeasurement (blood level: 4.5–8.4 ng/mL) prior to the experiment (Siemens Dimension EXL with LM Integrated Chemistry System; Siemens Healthineers; Germany). Treatments were administered for three consecutive days, then the mice were sacrificed. The removed kidneys were prepared for further analyses. In vivo experiments were conducted according to the guidelines of the Institutional Animal Care Facility and approved by the Institutional Ethical Review Board (PE/EA/801-7/2020 approval date: 16 September 2020) with official permissions (PEI/001/1733-2/2015 approval date: 14 October 2015).

### Human xenograft mouse model

RCC xenograft models were created by injecting 5 ×10^6^ tumour cells (786-O and A498) in 100 μl FBS-free cell culture media subcutaneously in 8-week-old male SCID mice (*n* = 20 for each cell line). Animals were kept the same as the C57BL/6 mice. The experiment started when tumours reached the palpable size after 2 further inoculations. Then the animals were randomised (simple method) into groups: #1: control IP (saline, IP, *n* = 5); #2: TAC (Advagraf; Astellas Pharma; 3 mg/kg; IP; *n* = 5); #3: RAPA (Rapamune; Pfizer; 1 mg/kg; *per os*; *n* = 5); #4: control *per os* (saline; *per os*; *n* = 5). Treatments were administered three times a week for 21 days. Tumour growth and the alteration of body weight were registered continuously. After 21-day treatment period, mice were sacrificed. The developed tumours, and kidneys were removed, then prepared for further analyses. The in vivo experiments were conducted according to the guidelines of the Institutional Animal Care Facility and approved by the Institutional Ethical Review Board (PE/EA/801-7/2020 approval date: 16 September 2020) with official permissions (PEI/001/1733-2/2015 approval date: 14 October 2015).

### Statistical analysis

For parametric data, t-test was used for the comparison of two groups, and one-way ANOVA with Tukey’s post hoc test was used when comparing more than two groups. Non-parametric data were analysed with Mann–Whitney U test. Chi-square test was used to compare categorical variables. Statistical analysis was performed using IBM SPSS Statistics software (SPSS Inc; USA (IL); 28.0.1.0). Experiments were evaluated from at least 3 parallel measurements.

## Results

### Analysis of mTOR activity in renal cell carcinoma and its correlation with immunosuppressive therapy

#### The characteristics of post-transplant renal cell carcinoma cohort

Between 2000 and 2015, a total of 2615 kidney transplantations (2561 kidney + 54 simultaneous pancreases + kidney) were performed at Semmelweis University. In our post-tx RCC collection (44 cases), our aim was to include an adequate representation of each subtype to conduct a more precise evaluation of the study (Table 1). The shift in subtype distribution was also observed in the Hungarian patient population, with higher occurrence of pRCCs. For the groups to be properly analysable, a sufficient number of cases had to be collected for each group, therefore the subtype-distribution of this cohort is not representative.

#### Activity of mTORC2 is significantly higher in post-transplant than in non-transplant renal cell carcinomas

**Fig. 1:**
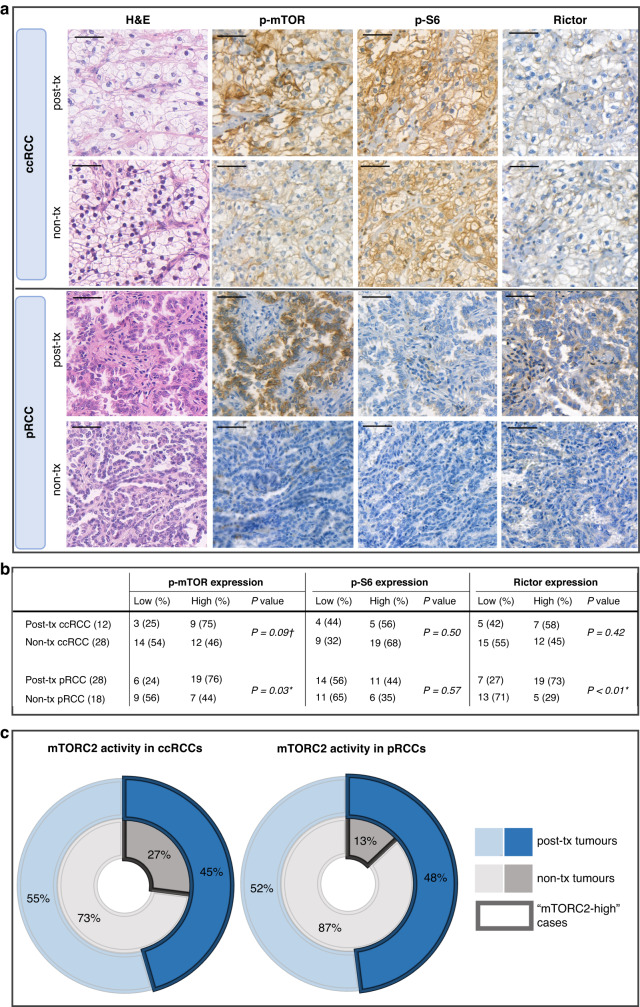
Higher mTORC1/C2 activity was observed in post-transplant compared to non-transplant renal cell carcinomas. mTORC1/C2 activity was higher in post-transplant (post-tx) compared to non-transplant (non-tx) papillary (pRCC) and clear cell (ccRCC) renal cell carcinomas. **a** Haematoxylin-eosin (H&E) and IHC stainings (p-mTOR, p-S6, Rictor, PTEN) were performed. DAB was used as a chromogen with haematoxylin counterstaining. Scale bars indicate 50 μm. **b** Expression of mTORC1/2 markers in post-tx and non-tx RCCs. Expressions are defined as deviations upper (high expression) or lower (low expression) from the median H-score value. *p < *0.05* was considered as statistically significant, ^†^*p* < *0.1*. **c** Case distribution regarding mTORC2 activity in post-tx and non-tx ccRCCs and pRCCs. “mTORC2-high” cases were defined based on the upper deviation from the median H-score values of both p-mTOR (the active form of the catalytic subunit of mTORC1 and mTORC2) and Rictor (scaffold protein of mTORC2).

The expression of p-mTOR, p-S6, and Rictor was analysed in post-tx RCCs using IHC and compared to non-tx RCC cases. Higher expression of p-mTOR was observed in post-tx ccRCCs compared to non-tx ccRCCs, although the difference was not statistically significant (*p* = *0.09*). The expression of p-mTOR and Rictor was significantly higher in post-tx pRCCs compared to non-tx pRCCs (*p* = *0.03 and p* < *0.01*, respectively) (Fig. 1A, B). Enhanced mTORC2 activity, as indicated by the increased expression of both p-mTOR (the active form of the catalytic subunit of mTORC1 and mTORC2) and Rictor (a scaffold protein of mTORC2), was detected in post-tx RCCs in contrast to non-tx RCCs in both subtypes (Fig. 1C).

#### mTOR activity increases in immunosuppressed end-stage renal disease compared to normal kidney

**Fig. 2:**
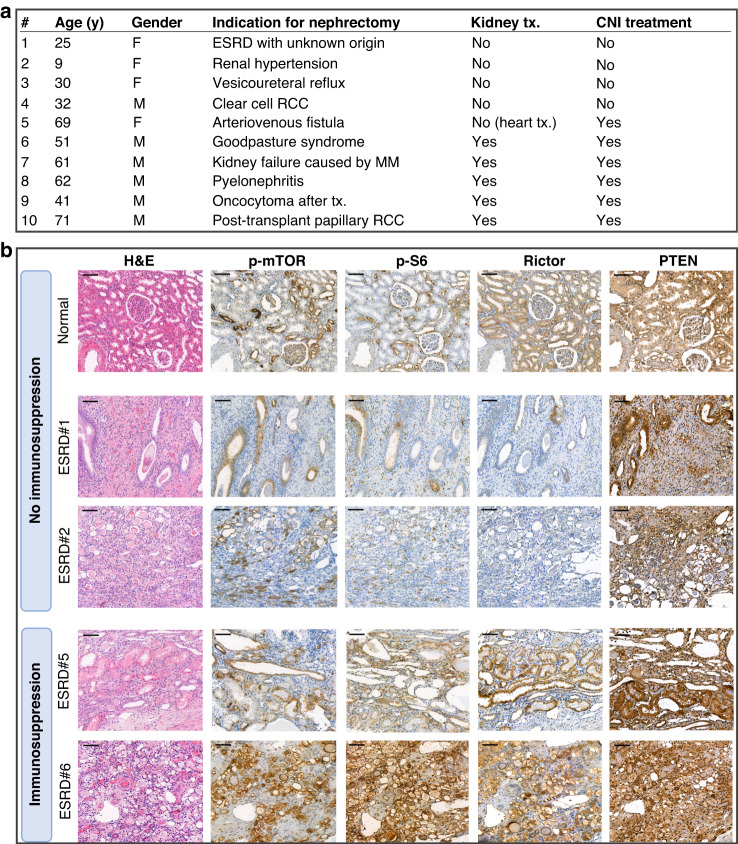
mTORC1/C2 activity elevated in patients with end-stage renal disease under the influence of calcineurin-inhibitor. Higher mTORC1/C2 activity was observed in patients with end-stage renal disease (ESRD) in response to CNI treatment compared to normal kidney and untreated ESRD patients; **a** Clinicopathological characteristics of ESRD patients; **b** Haematoxylin-eosin (H&E) and immunohistochemical stainings (p-mTOR, p-S6, Rictor, PTEN) were performed. DAB was used as a chromogen with haematoxylin counterstaining. Scale bars indicate 100 μm.

The expression and activity of mTOR complexes were studied in normal kidney tissues (donor kidneys that were not considered suitable for transplantation due to surgical reasons; *n* = 3) and kidneys with ESRD with or without CNI IS treatment (*n* = 10) using IHC (Fig. 2a). Weak expression of p-S6 and p-mTOR, along with high Rictor expression, was detected in normal tubular epithelial cells of non-malignant tissues. The expression of p-mTOR, p-S6 and Rictor was lower in ESRD kidneys without CNI treatment compared to normal kidneys. In contrast, KTRs treated with CNI IS therapy showed higher expression of p-mTOR, p-S6, and Rictor compared to both normal kidneys and kidneys of ESRD patients without CNI therapy, as shown on representative sections (Fig. 2b).

### In vitro effects of immunosuppressive treatments

#### Tacrolimus enhances mTOR activity, but does not affect proliferation of normal tubular epithelial cells

In vitro effects of TAC on mTOR activity and proliferation of normal tubular epithelial cells were analysed using an immortalised proximal tubule epithelial cell line (HK-2).

**Fig. 3:**
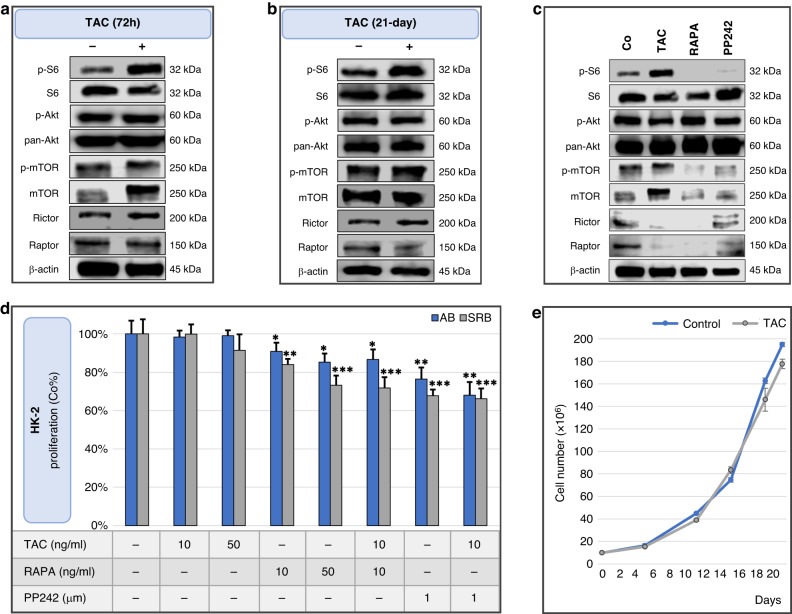
Tacrolimus tends to elevate the mTOR-activity of normal tubular epithelial cell line in vitro. Tacrolimus (TAC) elevates, while mTOR inhibitors decrease mTOR activity in vitro in normal tubular epithelial cell line (HK-2) (**a**, **b**) Western blot analyses of short- and long-term effect of TAC (10 ng/ml; 72 h and 21-day) on mTOR activity markers and (**c**) comparing the effects of TAC (10 ng/ml; 72 h), rapamycin (RAPA; 10 ng/ml, 72 h) and PP242 (1 μM; 72 h) on mTOR activity. Samples were derived from the same experiment, and the blots were processed in parallel. **d** Effects of tacrolimus, rapamycin and PP242 on proliferation of HK-2 cells (72 h). Used concentrations were indicated in the figure. Alamar Blue (AB) and Sulforhodamine B (SRB) proliferation assays were used. *p < *0.05*, **p < *0.01*, ***p < *0.001* (one-way ANOVA with Tukey’s post hoc test, the significance compared to the control). **e** Long-term (21-day) effect of tacrolimus (10 ng/ml) on HK-2 cell line. Proliferation was evaluated by cell counting. No cells were discarded during the experiment.

Based on Western blot image analyses, 72 h TAC treatment (10 ng/mL) significantly increased mTORC1 activity compared to the control, as indicated by the p-S6/S6 expression ratio [0.62 vs 0.45; *p* = *0.02*]. However, no increase in p-mTOR/mTOR [0.63 vs 1.41; *not significant* (*ns)*] and p-Ser473-Akt/Akt [0.41 vs 0.49; *ns*] ratios was observed in HK-2 cells (Fig. 3a). Prolonged treatment with TAC for 21 days slightly increased both p-S6/S6 [1.03 vs 0.66; *p* = *0.01*] and p-mTOR/mTOR [1.14 v 0.54; *p* = *0.06*] ratios, suggesting long-term activation of mTORC1. (Fig. 3b) Conversely, treatment with mTORC1 inhibitor (RAPA) and the dual mTORC1/2 inhibitor (PP242) decreased the p-mTOR/mTOR and p-S6/S6 ratios (Fig. 3c). Hence, TAC was suspected to increase mTOR activity, which could be reversed by RAPA or PP242 treatments.

The effects of TAC, RAPA and PP242 on proliferation of HK-2 cells were also studied using AB and SRB assays. TAC did not significantly affect proliferation during the 72 h treatment course, whereas RAPA and PP242 had a moderate anti-proliferative effect. Combined treatments (TAC + RAPA or PP242) and prolonged monotreatment with TAC (21 days) had no significant impact on proliferation (Fig. 3d, e).

#### Tacrolimus enhances mTORC activity and promotes proliferation of renal cell carcinoma cell lines

**Fig. 4:**
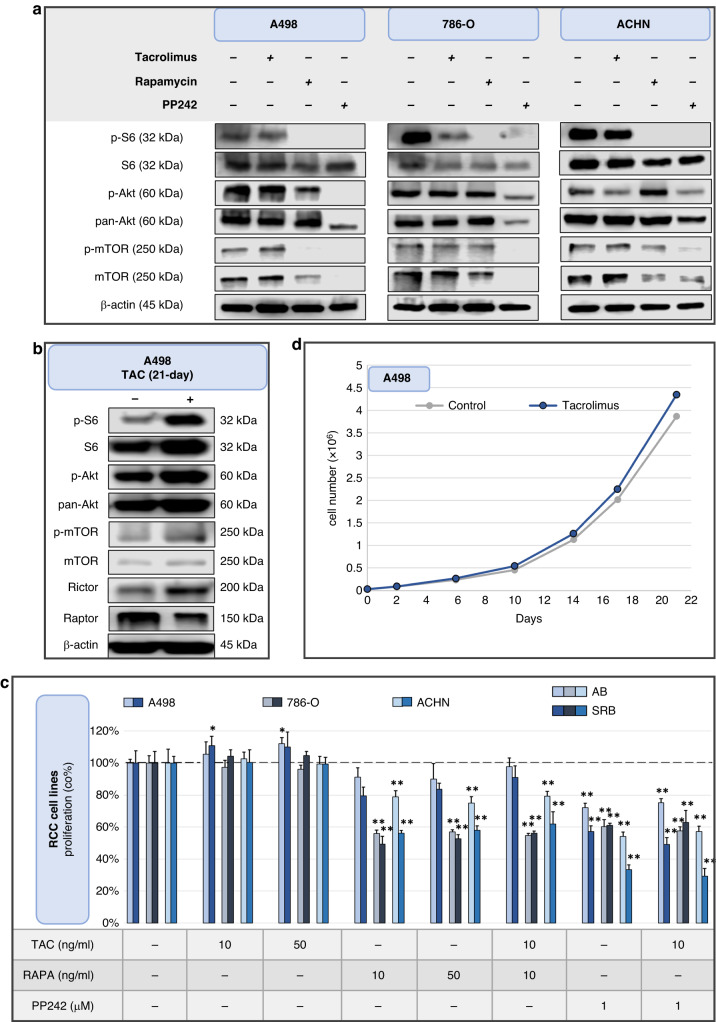
Tacrolimus elevates the mTOR activity and proliferation of renal cell carcinoma cell lines in vitro. Tacrolimus (TAC) enhances mTOR activity and promotes the proliferation of certain renal cell carcinoma cell lines, whereas mTOR inhibitors mostly attenuate these effects. **a**, **b** Western blot analyses of short- and long-term (72 h and 21-day) effect of TAC (10 ng/ml), rapamycin (RAPA, 10 ng/ml) and PP242 (1 μM) on mTOR activity markers. Samples were derived from the same experiment, and the blots were processed in parallel. **c** Effects of TAC, RAPA and PP242 on proliferation of HK-2 cells (72 h). Used concentrations were indicated in the figure. *p < *0.05*, **P < *0.01* (one-way ANOVA with Tukey’s post hoc test, the significance compared to the control). **d** Long-term (21-day) effect of TAC (10 ng/ml) on A498 cell line. Proliferation was evaluated by cell counting. No cells were discarded during the experiment. *p* = *0.07* (paired t-test).

72 h TAC treatment modestly increased the p-mTOR/mTOR ratio in all investigated RCC cell lines (A498 [1.71 vs 0.61; *p* = *0.04*], 786-O [1.63 vs 0.74; *ns*], ACHN [1.77 vs 0.61; *ns*]), indicating a slight increase in mTOR activity. This increase was confirmed through β-actin-normalised densitometry evaluation (although the results were not always statistically significant due to the small sample size), while visually detectable differences were only found in case of A498. RAPA and PP242 generally decreased mTOR activity. After RAPA treatment, p-S6 (related to mTORC1 activity) expression was nearly undetectable in all cell lines. Additionally, RAPA had no significant influence on mTORC2 activity (p-Ser473-Akt/pan-Akt) (Fig. 4a). Furthermore, the changes in mTOR activity were examined in A498 cell line following long-term (21 days) TAC treatment. Increased pS6/S6 [0.81 vs 0.25; *p* = *0.04*], p-Ser473-Akt/pan-Akt [1.21 vs 0.66; *ns*] and p-mTOR/mTOR [3.90 vs 1.30; *p* = *0.06*] ratios were observed, indicating the activation of both mTORC1 and mTORC2 during prolonged TAC treatment (Fig. 4b).

Consistent with the previously demonstrated increased mTOR activity, low- and high-dose TAC treatments (72 h) slightly induced proliferation in A498 cells (*p* < *0.05*), which was confirmed by AB and SRB assays. Moreover, A498 RCC cell line exhibited resistance to RAPA, while the other two RCC cell lines showed sensitivity in 72 h in vitro experiments. The dual inhibitor treatment (PP242) suppressed the proliferation more effectively in all cell lines, as expected (Fig. 4c). Due to the observed minor increase in proliferation during the 72 h TAC treatment in the A498 cell line, the study was repeated with a long-term (21 days) treatment course. Cell counts were manually performed during subculturing, and the same TAC concentration was maintained throughout the treatment. A 10% increase in proliferation was observed compared to untreated cultures, although it did not reach statistical significance (*p* = *0.07* was considered as a trend) (Fig. 4d).

### Tacrolimus induces mTOR-activity and promotes proliferation in vivo in ischaemia-reperfusion mouse model and A498 human xenograft model

**Fig. 5:**
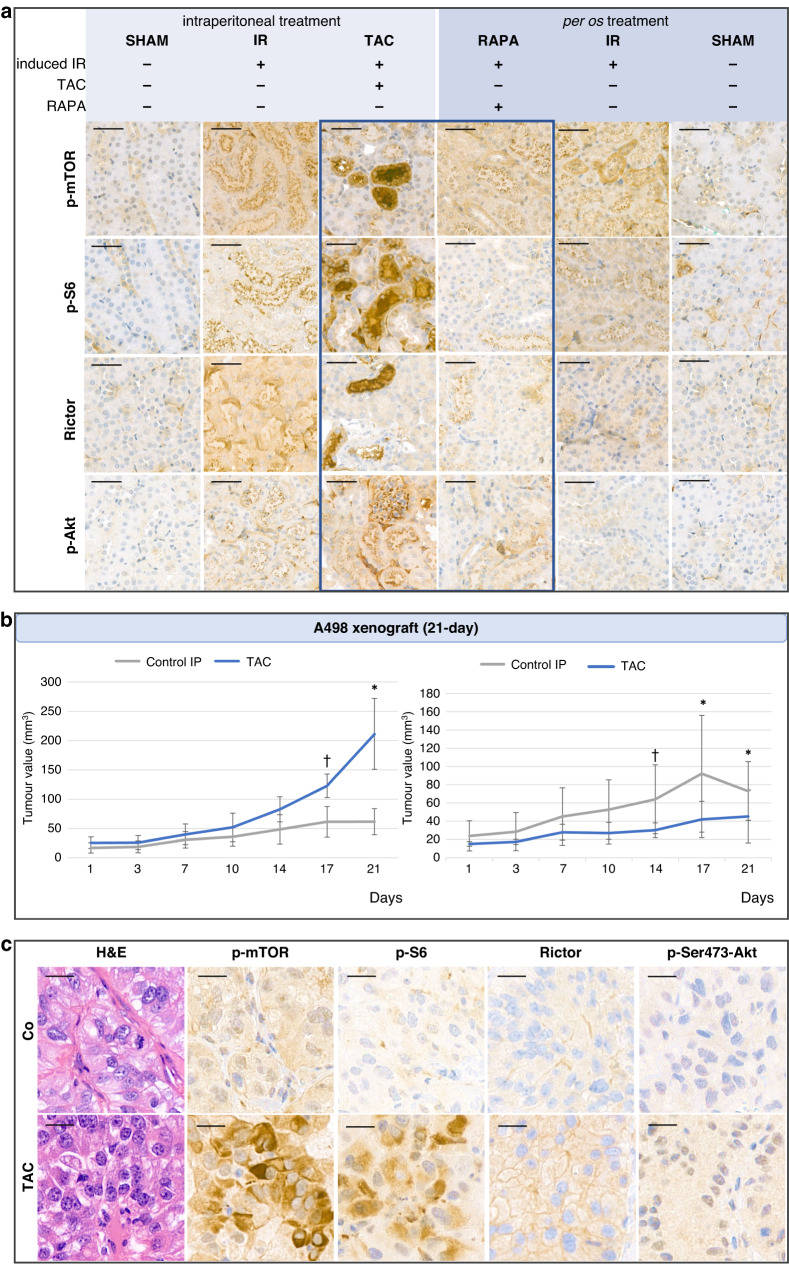
Tacrolimus elevates the mTOR-activity of ischaemic kidneys in mice, promotes proliferation and increases mTOR-activity of renal cell carcinoma xenograft in vivo. Tacrolimus (TAC) induces mTOR activation and promotes proliferation in vivo. **a** Tacrolimus (TAC) increases mTORC1/C2 activity, while rapamycin (RAPA) has a moderate decreasing effect in induced ischaemic kidneys of mice in vivo. IHC stainings (mTORC1: p-mTOR, p-S6; mTORC2: p-mTOR, Rictor, p-Ser473-Akt) were performed on removed kidneys (SHAM = placebo surgery; IR = induced ischaemia). DAB was used as a chromogen and haematoxylin counterstaining was performed on IHC stainings. Scale bars indicate 50 μm. **b** Tumour growth of TAC-treated (3 mg/kg, 3/week, 21 days) and RAPA-treated (1.5 mg/kg, 3/week, 21-day) A498 xenografts. ^†^*p* < *0.1*, *p < *0.05* (paired t-test). **c** IHC stainings (mTORC1: p-mTOR, p-S6; mTORC2: p-mTOR, Rictor, p-Ser473-Akt) were performed on removed A498 xenograft tumours. DAB was used as a chromogen and haematoxylin counterstaining was performed on IHC stainings. Scale bars indicate 20 μm.

Renal ischaemia-reperfusion (IR) mouse model was created using C57BL/6 mice. After three consecutive days of treatment (TAC or RAPA), the kidneys were removed and IHC stainings (p-mTOR, p-S6, Rictor, p-Akt) were performed. Increased expression of p-mTOR, p-S6 and Rictor was observed in IR groups (induced ischaemia, placebo treatment) compared to the SHAM groups (placebo surgery, placebo treatment). In TAC-treated animals’ kidney, particularly high mTORC1 activity (p-mTOR, p-S6) was observed in some tubules. Additionally, mTORC2-activity (p-mTOR, Rictor, p-Akt) showed a similar pattern, but its expression was more moderate. In RAPA-treated animals, a slight decrease in mTOR-activity was observed in the ischaemic kidney compared to the untreated (only IR) group (Fig. 5a).

Human xenograft mouse model was generated using the A498 cell line and SCID mice. A 21-day treatment with TAC (3 mg/kg; 3x/week; *n* = 5) enhanced tumour growth (average tumour size of control and TAC groups were 61.34 ± 22.37 mm^3^/0.6 ± 0.27 g and 211.17 ± 60.45 mm^3^/1.25 ± 0.64 g, respectively). In contrast, RAPA treatment (1.5 mg/kg; 3x/week; *n* = 5) inhibited tumour growth, as expected (average tumour size of control and RAPA groups were 85.53 ± 18.34 mm^3^/1.18 ± 0.36 g and 45.17 ± 29.16 mm^3^/0.32 ± 0.18 g, respectively) (Fig. 5b). IHC stainings showed increased mTORC1 activity: significantly increased p-mTOR, p-S6 and a slight increase in Rictor and p-Ser473-Akt were observed in TAC treated tumours (Fig. 5c). These results are consistent with the previously described in vitro tumour-growth-promoting effects of TAC in A498 cell line. This effect appeared to be more dependent on the tumour type rather than being correlated to immunosuppression in xenografts, as the tumour growth of 786-O (another ccRCC cell line) was not affected by TAC in the in vivo xenograft model (data not shown).

## Discussion

As chronic kidney disease (CKD) progresses, the kidney undergoes a slow structural and functional damage, leading to fibrosis and loss of its function. Fibrosis is a process closely associated with inflammation and naturally plays a protective role against injuries as a part of the physiological wound-healing mechanism. Failure in wound-healing leads to continuous tissue regeneration, resulting in excessive extracellular matrix (ECM) accumulation and pathological fibrosis. Pathological fibrosis replaces the renal parenchyma, leading to irreversible renal failure and ESRD [39], where tissue damage triggers chronic inflammation, characterised by the infiltration of various inflammatory cells [40]. Inflammatory cells release transforming growth factor beta 1 (TGF-ß1), which stimulates the synthesis of ECM proteins, contributing to renal fibrosis [41, 42]. It is a vicious circle that lasts until the kidney completely loses its function.

In case of kidney transplantation, the non-functioning kidneys of recipients are usually left in the abdominal cavity and have already progressed to ESRD before transplantation. Chronic inflammatory and fibrotic processes in these kidneys result in tumour-promoting cellular environment and signalling failures [43, 44].

IS treatment is required after transplantation of which TAC remains still an integral part today. Since the approval of TAC (1994), IS regimen has remained almost unchanged. Some alternative IS agents, such as rapalogs are becoming increasingly popular, but TAC remains the cornerstone of maintenance therapy.

In our experimental research, it was observed that TAC tends to increase tumorigenic mTOR activation, confirmed both in vitro and in vivo. TAC increased mTORC1/C2 activation in the investigated cell lines in vitro, while in vivo experiments using an IR model demonstrated increased mTORC1/C2 activation in the ischaemic kidneys of mice. Additionally, TAC enhanced both the mTOR activity and the tumour growth of A498 xenografts. The effects of TAC on mTOR activation and proliferation appears to be influenced by the cell type and the ischaemic or fibrotic conditions in the cellular environment. Importantly, mTOR activation may contribute to the elevated tumour risk observed in KTRs who receive TAC as part of their immunosuppressive therapy.

In addition to its main CNI effect, TAC is also capable of stimulating the expression of TGF-β in a dose-dependent manner through the ERK pathway [45, 46]. Activated TGF-β could stimulate various downstream pathways, including the main/canonic (SMAD) and alternative pathways (MAPK, ERK1/2, PI3K/AKT and JNK), contributing to EMT promotion, ECM accumulation, immune invasion and tumorigenesis [47, 48]. Furthermore, recent studies suggest that TAC-based IS regimens may also affect cancer progression through mTOR pathway activation. It has been reported that CNI treatment activates the proto-oncogene Ras [49] and promotes the phosphorylation of PRAS40 (negative regulator of mTORC1) to increase mTOR activity [50]. However, inhibitory effect of TAC on mTORC1 has also been observed in studies related to insulin resistance, but these effects were observed in pancreatic islets and not in tumour cells [51]. These suggest that the effects of TAC on mTOR activation may vary depending on the cell type and that TAC could positively influence tumorigenic mTOR activation in certain cancer or pre-cancer cells. These complex effects of inflammation, fibrosis and molecular mechanisms of TAC may contribute together to the development of post-tx RCCs, which needs further clarification.

The PI3K/Akt/mTOR pathway has a fundamental role in renal physiology, and its inhibitors are used both as IS and antitumor agents. In case of a patient developing a tumour during IS therapy, the use of mTOR inhibitors should be considered. However, it may be important to assess the mTOR activity of tumour cells before initiating mTOR inhibitor therapy. Although rapalogs are generally used to reduce the risk of tumours after transplantation [52, 53], there is a new study from 2022 questioning the effectiveness of early conversion to mTOR inhibitor-based IS regimens in reducing tumour incidence [54].

While there is limited evidence regarding the preventive role of mTORC1 inhibitors as IS agents against post-tx malignancies, our findings emphasise the involvement of both mTORC1 and mTORC2 in the pathobiology of post-tx RCCs. Our study revealed a significantly higher occurrence of tumours with mTORC2 hyperactivation among post-tx tumours compared to non-tx RCCs, indicating the role of mTORC2 activation in the development of post-tx tumours. Increased mTORC2 activity has been associated with worse prognosis in various types of tumours, such as endometrial cancer [55], gastric cancer [56], colon cancer [57] and small cell lung cancer [58]. Higher incidence of mTORC2 hyperactivation among post-tx tumours, along with the role of mTORC2 in migration and metabolism may explain the increased cancer-specific mortality observed in recipients with cancer compared to non-tx patients [59].

We observed an increased level of mTORC2 in the ischaemic kidney, indicating its regulatory role in the healing or fibrotic processes of injured kidney, where maintaining a delicate balance between cell death, proliferation and survival is crucial. This regulatory network is highly vulnerable, where elevated activity of Rictor and mTORC2 could potentially lead to imbalanced cell growth or excessive proliferation. Although rapalog-based IS therapy may not necessarily improve tumour incidence, combining it with TAC should be considered. Administration of everolimus (an mTORC1 inhibitor) has been shown to effectively suppress the activation of kidney fibroblasts and inhibit the expression of ECM proteins [60]. This combined use of RAPA and TAC may potentially minimise the fibrosis-promoting effects of TAC and the severe adverse effects of RAPA. On the other hand, further investigation is needed to explore the use of dual mTORC1/C2 inhibitors as IS agents, as it holds promising potential in the opportunity in prevention or treatment of post-transplant RCCs. However, specific clinical trials in this direction have not been conducted yet.

In conclusion, our experimental findings firstly indicate that the specific ischaemic and fibrotic environment of non-functioning end-stage kidneys may contribute to the development of post-tx RCCs through elevated mTOR activity. In correlation with these, TAC tends to increase tumorigenic mTORC1/C2 activation and promotes proliferation in a cell-type dependent manner in epithelial and RCC cell lines, induced ischaemic kidneys, and RCC xenograft tumours as well. Elevated mTOR activity is commonly observed in various types of cancer and is also increased in the ischaemic and fibrotic environment of non-functional kidneys in ESRD patients. TAC tends to further enhance this already elevated mTOR activity, which can contribute to tumour development in the remaining kidneys of KTRs during IS therapy after transplantation.

## Data Availability

All the data obtained or examined during this study has been incorporated into this published article or is available on request from the corresponding author.
